# Bone morphogenetic protein 4 promotes the survival and preserves the structure of flow-sorted Bhlhb5+ cochlear spiral ganglion neurons *in vitro*

**DOI:** 10.1038/s41598-017-03810-w

**Published:** 2017-06-14

**Authors:** Muhammad Waqas, Shan Sun, Chuanyin Xuan, Qiaojun Fang, Xiaoli Zhang, Irum-us Islam, Jieyu Qi, Shasha Zhang, Xia Gao, Mingliang Tang, Haibo Shi, Huawei Li, Renjie Chai

**Affiliations:** 1 Key Laboratory for Developmental Genes and Human Disease, Ministry of Education, Institute of Life Sciences, Southeast University, Nanjing, 210096 China; 2 Department of Otolaryngology Head and Neck Surgery, Zhongda Hospital, Southeast University, Nanjing, 210096 China; 3 Co-innovation Center of Neuroregeneration, Nantong University, Nantong, 226001 China; 4 Department of Otorhinolaryngology, Affiliated Eye and ENT Hospital, State Key Laboratory of Medical Neurobiology, Fudan University, Shanghai, 200031 China; 5 Key Laboratory of Hearing Medicine of NHFPC, Shanghai, 200031 China; 6 Department of Otolaryngology, Affiliated Drum Tower Hospital of Nanjing University Medical School, Nanjing, 210008 China; 7 Department of Otorhinolaryngology Head & Neck Surgery, The Sixth People’s Hospital Affiliated to Shanghai Jiao Tong University, Shanghai, 200233 China; 8 Institutes of Biomedical Sciences, Fudan University, Shanghai, 200032 China; 9 Shanghai Engineering Research Centre of Cochlear Implants, Shanghai, 200031 China; 10 The Institutes of Brain Science and the Collaborative Innovation Center for Brain Science, Fudan University, Shanghai, 200032 China; 11 Department of Biotechnology, Federal Urdu University of Arts, Science and Technology, Gulshan-e-Iqbal campus, Karachi, Pakistan

## Abstract

SGNs are the primary auditory neurons, and damage or loss of SGNs leads to sensorineural hearing loss. BMP4 is a growth factor that belongs to the TGF-β superfamily and has been shown to play a key role during development, but little is known about its effect on postnatal cochlear SGNs in mice. In this study, we used the P3 Bhlhb5-cre/tdTomato transgenic mouse model and FACS to isolate a pure population of Bhlhb5+ SGNs. We found that BMP4 significantly promoted SGN survival after 7 days of culture. We observed fewer apoptotic cells and decreased expression of pro-apoptotic marker genes after BMP4 treatment. We also found that BMP4 promoted monopolar neurite outgrowth of isolated SGNs, and high concentrations of BMP4 preserved the number and the length of neurites in the explant culture of the modiolus harboring the SGNs. We showed that high concentration of BMP4 enhanced neurite growth as determined by the higher average number of filopodia and the larger area of the growth cone. Finally, we found that high concentrations of BMP4 significantly elevated the synapse density of SGNs in explant culture. Thus, our findings suggest that BMP4 has the potential to promote the survival and preserve the structure of SGNs.

## Introduction

Spiral ganglion neurons (SGNs) are the primary auditory neurons responsible for delivering the sound signals from the hair cells to the central nervous system via the cochlear nerve^1^. In mammals, the loss of sensory hair cells and the degeneration of SGNs is permanent because these cells do not spontaneously regenerate^2, 3^. Damage to the SGNs along with their associated synapses is an important cause of sensorineural hearing loss and auditory neuropathy, which is a hearing disorder caused by poor auditory perceptual capabilities^4, 5^. Therefore, the survival and structure of SGNs is important for maintaining proper hearing function. Some of the nerve growth factors such as BDNF (Brain Derived Neurotrophic Factor) and NT3 (Neurotrophin-3) have been thoroughly studied and are well known to promote SGN survival after hair cell damage both *in vivo* and *in vitro* ^6–8^.

Bone morphogenetic protein 4 (BMP4) is a member of the transforming growth factor-β (TGF-β) superfamily^9^. Members of the TGF-β family are involved in multiple biological events such as neural induction^10^, tissue patterning^11^, the epithelial–mesenchymal interactions underlying organogenesis^12^, lineage selection^13^, and the creation of stem cell “niches” in developing and adult organs^14, 15^. BMP4 is synthesized as a large precursor and exerts its biological function by interacting with membrane-bound receptors belonging to the serine/threonine kinase family to initiate intracellular cascade events. These receptors include bone morphogenetic protein receptors I (BMPRIA, BMPRIB), II (BMPRII), and III (TbetaRIII)^16, 17^. BMPs act as antagonists to neural differentiation during the early developmental stages in which progenitor cells transform into mesoderm or ectoderm, but several studies have also identified a role for BMPs in the specification of autonomic and sensory neurons from progenitors of the neural crest^18–21^. BMPs also promote the differentiation of glial cells from adult stem cells in the CNS^14, 22^. Transgenic over-expression of BMP4 increases astrocyte differentiation and inhibits oligodendrocyte formation^23, 24^. BMP4 is also expressed during the delamination of neural progenitors from the cranial placode and in the developing otocyst, where it participates in the development of inner ear hair cells and SGNs^25, 26^. Moreover, BMP4 signaling modulates the generation of hair cells in cultured chick otocysts^27^, and it is required for patterning of the sensory and nonsensory tissue^28^ and is essential for cochlear sensory formation and hair cell differentiation in the mammalian cochlea^29^.

BMP4 is a well-known factor that plays important roles during the development of the inner ear^26, 28, 30^. BMP4 is expressed as early as embryonic day (E)9.0 in the otic placode, and its expression persists until postnatal day (P)1 in the mesenchymal and sensory epithelium of neonatal mice^9, 31–33^. A crucial role of BMP4 in auditory neurogenesis is indicated by the expression of BMP4 and intracellular downstream signaling molecules (SMADs, Mammalian homologue of Drosophila Mad and C. elegans Sma) along with other transcription factors such as neurogenin-1(Neurog1) and neurogenic differentiation factor (Neurod1) in a distinct temporal pattern in vestibuloacoustic ganglia^26, 34–36^. In *in vitro* culture systems, exogenous BMP4 promotes the survival of dissociated SGNs isolated from the postnatal mouse cochlea^37^. Complete absence of the BMP4 gene results in embryonic death of mice between E6.5 and E9.5, which is prior to the development of the inner ear sensory epithelium^38^. BMP4 heterozygous+/− mice are viable but have elevated hearing thresholds, and adult BMP4+/− mice display a circling phenotype, indicative of an inner ear defect^38^. No structural abnormalities are observed in the cochlear hair cells and stereocilia of these heterozygous mice, but the number of SGNs decreases in the spiral ganglion region, which might be the reason for the partial hearing loss in these mice^33^. These studies indicate that BMP4 plays a critical role during the development of SGNs, but little is known about how it affects the survival and structure of mature SGNs.

The most established way to examine the effects of BMPs and other neurotrophic compounds on SGNs is through cell culture experiments that involve harvesting and digesting the modiolus that harbors the neuronal and non-neuronal cells to be cultured^37, 39–41^. The main limitation with dissociated cell culture is the presence of a large population of non-neuronal cells such as glial cells and fibroblasts, and these non-neuronal cells proliferate at higher rates than neuronal cells during *in vitro* culture^41, 42^. The increased proliferation of these cells limits the long-term cultivation of SGNs due to cellular overgrowth, and the presence of non-neuronal cells introduces an uncontrolled variability in the *in vitro* culture experiment. Thus, it is possible that the effects of exogenous treatment of different growth factors on SGN survival or neurite outgrowth might be affected by the endogenous secretion of molecules from the non-neuronal cells. Until now, there have been no published studies showing the isolation of a pure population of SGNs from the modiolus before culturing *in vitro*, which could eliminate the influence of the non-neuronal cells. Here, we isolated a pure population of SGNs by taking advantage of Bhlhb5-cre/tdTomato transgenic mice. Bhlhb5 is an olig-related basic helix-loop-helix transcription factor that is the key factor in neural specification and is required for the development of SGNs, and Bhlhb5 is specifically expressed in the SGNs in the inner ear^43^.

In the present study, we successfully isolated a pure population of Bhlhb5+ SGNs via flow-activated cell sorting (FACS) and determined the exogenous effects of BMP4 on the survival and structure of both flow-sorted Bhlhb5+ SGNs and the SGNs in cochlear explant cultures. We found that BMP4 promotes the survival of Bhlhb5+ SGNs and protects the Bhlhb5+ SGNs against apoptosis. High concentrations of BMP4 also enhance neurite growth and elevate the synapse density of SGNs in an *in vitro* culture system. In conclusion, we have shown that *in vitro* treatment with BMP4 promotes the survival and preserves the structure of cochlear SGNs in culture, and this suggests an additional role for BMP4 in the development of the inner ear.

## Materials and Methods

### Animals

Bhlhb5-cre mice [13] (kindly gifted from Dr. Lin Gan, University of Rochester, NY) and Rosa26-tdTomato reporter mice (The Jackson Laboratory, Stock #007914) were housed in groups of 4 to 5 and inspected daily for infections or abnormal behaviour. They were allowed free access to both food and water for the entire duration of the experiments and were housed under controlled temperature (22–25 °C) and humidity with 12 h alternate light-dark cycles. All animal procedures were performed according to the protocols approved by the Animal Care and Use Committee of Southeast University and were consistent with the National Institutes of Health Guide for the Care and Use of Laboratory Animals. All efforts were made to minimize the number of animals used and to prevent their suffering.

### *In vivo* labeling of Bhlhb5+ SGNs

Bhlhb5 is specifically expressed in SGNs in the inner ear. Bhlhb5-cre mice were crossed with Rosa26-tdTomato mice (homozygous) to label the Bhlhb5+ SGNs in the modiolus with tdTomato. Bhlhb5-cre+/tdTomato+ mice were sacrificed at P3, and the modiolus harboring the SGNs was dissected and subjected to cryosection and immunostaining.

### Isolation of Bhlhb5+ SGNs via flow cytometry

P3 Bhlhb5-cre +/tdTomato+ mice were used for the experiment. The dissection was performed as described previously (9, 15) with minor modification. Briefly, mice were decapitated and their skulls were opened mid-sagittally. Both of the temporal bones were immediately cut off and transferred to ice cold Hank’s balanced salt solution (HBSS 1X, Invitrogen Life Technologies). The cochlear capsule was opened and the membranous labyrinth was removed from the modiolus under a dissecting microscope. The stria vascularis and the organ of Corti were carefully removed, then the modiolus harboring the SGNs was collected in a separate tube and trypsinized with prewarmed 0.125% trypsin/EDTA (Invitrogen) at 37 °C for 10 min. The reaction was then terminated by adding soybean trypsin inhibitor (10 mg/ml, Worthington Biochem). Cells were separated by mechanical trituration using blunt tips and pipetting up and down 80–100 times. Suspended cells were passed through a 40 µm cell strainer (BD Biosciences). Dissociated cells from the SGN pool were then sorted on a BD FACS Aria III using the red channel. Immunostaining and qPCR was performed to evaluate the purity of the flow-sorted cells.

### Genotyping and real-time quantitative PCR

Transgenic mice were genotyped using genomic DNA from tail tips by adding 90 µl of 50 mM NaOH, incubating at 98 °C for 20–50 min, and adding 10 µl of 1 M HCl. The genotyping primers were as follows: Bhlhb5-cre: (F) GGG ATT GGA CTC AGA GGC GGT AGC, (R) GCC CAA ATG TTG CTG GAT AGT; tdTomato: wild-type (F) AAG GGA GCT GCA GTG GAG T, (R) CCG AAA ATC TGT GGG AAG TC; mutant (F) GGC ATT AAA GCA GCG TAT C; (R) CTG TTC CTG TAC GGC ATG G.

For real-time quantitative PCR (qPCR), the Cells-to-cDNA II kit (Ambion; AM 1722) was used to extract total RNA and reverse transcribe it into cDNA using oligo(dT) primers. The SYBR Premix Ex Taq (Tli RNase H Plus) kit (TaKaRa) was used to perform qPCR on a Bio-Rad C1000 Touch thermal cycler. Each qPCR reaction was performed in triplicate with β-actin as the reference endogenous gene, and all reactions were analyzed using the ΔΔCT method. The following primers were used in the qPCR experiment: Tuj1: (F) TGA AGT CAG CAT GAG GGA GAT CG, (R) TGC CAG CAC CAC TCT GAC CAA; Sox2: (F) GCG GAG TGG AAA CTT TTG TCC, (R) CGG GAA GCG TGT ACT TAT CCT T; β-actin: (F) GGC TGT ATT CCC CTC CAT CG, (R) CCA GTT GGT AAC AAT GCC ATG T; Casp3: (F) GGA GCA GCT TTG TGT GTG TG, (R) CTT TCC AGT CAG ACT CCG GC; Casp8: (F) GCT GTA TCC TAT CCC ACG, (R) TCA TCA GGC ACT CCT TT; Casp9: (F) GGA CCG TGA CAA ACT TGA GC, (R) TCT CCA TCA AAG CCG TGA CC; Apaf1: (F) TGT GTG AAG GTG GAG TCA AGG, (R) CCT CTG GGG TTT CTG CTG AA; Bax: (F) CGT GGT TGC CCT CTT CTA CT, (R) TTG GAT CCA GAC AAG CAG CC; and Bcl2: (F) TGA CTT CTC TCG TCG CTA CCG, (R) GTG AAG GGC GTC AGG TGC AG.

### Culture of flow-sorted Bhlhb5+ SGNs

Sorted Bhlhb5+ SGNs were cultured to a density of 10 cells/µl on laminin-coated plates using DMEM/F12 medium supplemented with N2 (1:100 dilution, Invitrogen) and B27 (1:50 dilution, Invitrogen). At the same time, various concentrations of BMP4 or Noggin were added to the media. The cells were incubated at 37 °C and cultured for 7 days.

### Explant culture

P3 mice were used for the explant culture, and the dissection procedures were performed as described above. In order to keep the sample consistent between the groups, the middle turn of the cochlea was selected for all explant culture experiments. After dissection, the modiolus harboring the SGNs was transferred onto a 10 mm glass coverslip precoated with CellTak (BD Biosciences) and cultured in DMEM/F12 medium supplemented with N2 (1:100 dilution, Invitrogen) and B27 (1:50 dilution, Invitrogen) combined with the different concentrations of BMP4 or Noggin and transferred to the incubator. All of the explants were then cultured for 7 days at 37 °C, 5% CO_2_, and 95% humidity.

### Immunostaining and image acquisition

Cultured SGNs were fixed in 4% PFA for 1 hour at room temperature followed by blocking of nonspecific binding sites with PBS (pH 7.4) containing 5% donkey serum, 0.1% Triton X100, 1% bovine serum albumin (BSA), and 0.02% sodium azide (NaN_3_) for 1 h at room temperature. The SGNs were then incubated overnight with primary antibodies diluted in the same blocking solution at 4 °C. The next day, the SGNs were washed three times with PBS and incubated with the secondary antibody (Invitrogen) for 1 h at room temperature. Cells were then washed three times with PBS and mounted in antifade fluorescence mounting medium (DAKO). The following antibodies were used in this study: anti-NeuN antibody (Cell Signaling Technology) was used as a neuronal marker; anti-Tuj1 antibody (Neuromics) was used to label the neurofilament and nerve endings; anti-PSD95 antibody (Millipore) was used to label the postsynaptic membrane; anti-Sox2 antibody (Santa Cruz Biotechnology) was used to label the glial cells because Sox2 plays an essential role in maintaining the pluripotency and self-renewal of undifferentiated embryonic stem cells; and anti-phalloidin antibody (Sigma-Aldrich) was used to identify the actin cytoskeleton network. Apoptosis in SGNs was examined by DNA fragmentation with a TUNEL staining kit (Roche) according to the manufacturer’s instructions. A Zeiss LSM 710 confocal microscope was used to capture images, and the images were analyzed using ImageJ (NIH) and Photoshop CS4 (Adobe System).

### Data Analysis

After immunostaining, the SGNs were assessed for the effects of BMP4 or Noggin by counting the number of surviving SGNs and measuring their neurite outgrowth. Neuronal morphology was scored as monopolar, bipolar, multipolar, or no neurites. All neurites longer than 15 μm (approximately two times longer than the average diameter of the neurons) were counted and measured. Bipolar neurons were scored solely based on the number of neurites with the appropriate length, but not based on the location of neurites around the cell soma, i.e., two neurites on the same side of the cell body or two on opposite sides were both counted as bipolar. The percentages of different morphologies were calculated from the total numbers of neurons that were scored unambiguously in each well. Multipolar neurons and neurons with no neurites made up less than 10% of the total numbers of neurons.

Nerve fibers were classified as F1 (the longer fiber of a bipolar neuron), F2 (the shorter fiber of a bipolar neuron), or Fm (the fiber from a monopolar neuron). Each neuron was digitally photographed on a Zeiss LSM 710 confocal microscope, and the fibers in the resulting image were measured on the computer monitor with the aid of the ImageJ (NIH) and Photoshop CS4 (Adobe System) software.

For explant culture, neurite outgrowth from the ganglia was evaluated in terms of the number and length of the processes that stained positive for Tuj-1. Images were taken at different focal planes if required to visualize all of the neurites. The number of surviving neurons in each explant was normalized to the number of neurites in each explant. For every explant, the length of the neurites was determined by marking the linear distance from the ganglion edge to the tip of the growth cone of the processes.

### Statistical analysis

For each condition, at least three individual experiments were performed. Data are presented as mean ± SD. The Prism software (GraphPad) was used to perform all statistical analyses and to plot the figures. A two-tailed, unpaired Student’s *t*-test was performed when comparing two groups, and a one-way ANOVA followed by a Dunnett’s multiple comparisons test was used when comparing more than two groups. All *p*-values < 0.05 were considered statistically significant.

## Results

### *In vivo* expression of tdTomato in Bhlhb5-cre +/tdTomato+ mice

**Figure 1:**
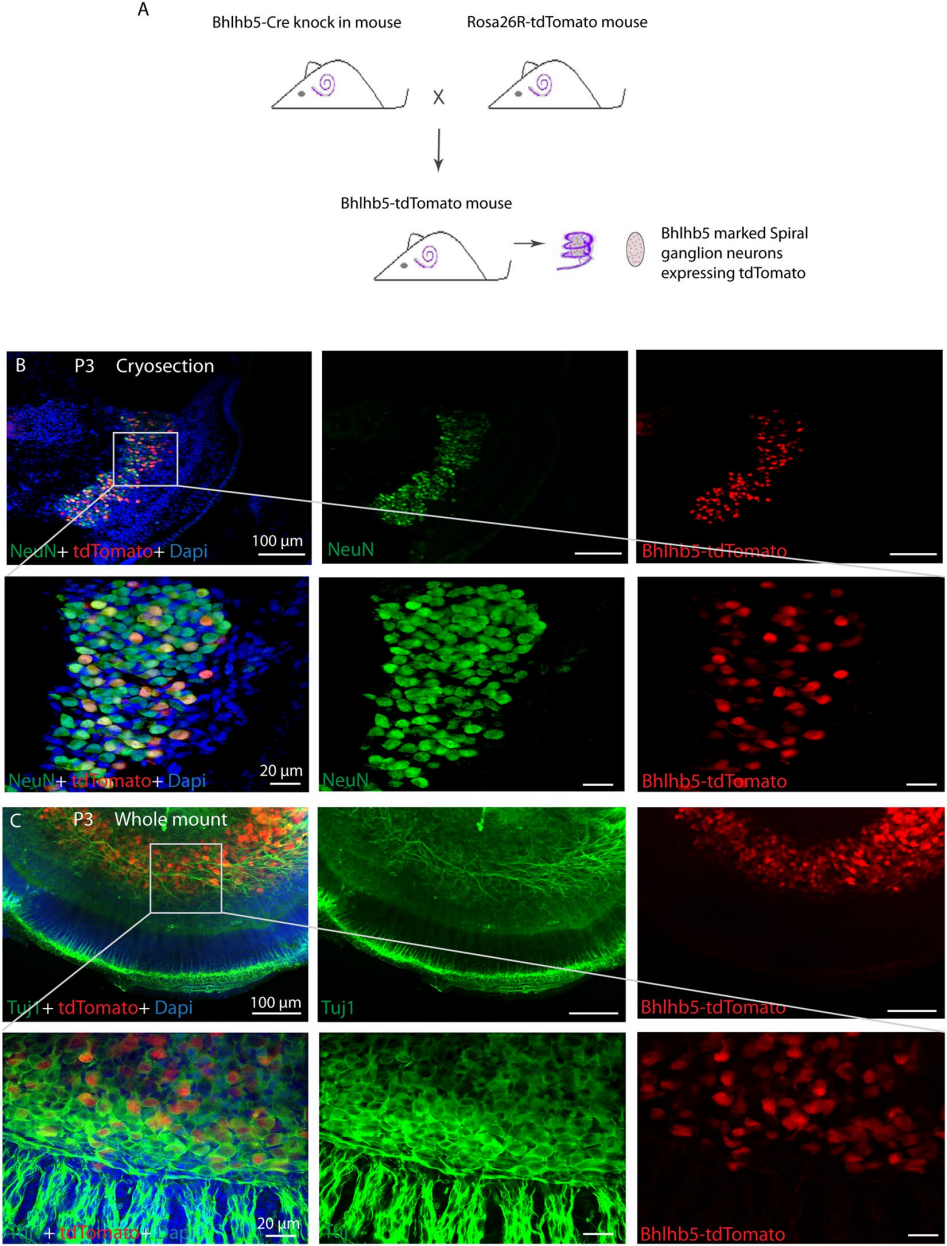
*In vivo* lineage tracing of Bhlhb5+ SGNs in the postnatal cochlea: (**A**) Bhlhb5-cre knock-in mice were crossed with the Rosa26-tdTomato reporter strain. The resulting littermates were genotyped, and the cochleae of Bhlhb5-tdTomato+ mice were examined at P3. (**B** and **C**) Traced tdTomato/NeuN+ and tdTomato/Tuj1+ SGNs were observed in cryosections and whole mounts of the modiolus.

In order to determine the expression of Bhlhb5 in the SGNs, we first labeled the Bhlhb5+ SGNs by crossing Bhlhb5-cre mice with the Rosa26-tdTomato reporter strain (Fig. 1A). Cochleae from P3 mice were harvested and examined by cryosectioning and whole-mount staining. We found that the tdTomato+ cells were 100% co-labeled with the neuronal marker NeuN (Fig. 1B), suggesting that tdTomato had specifically labeled the SGNs. However, not every NeuN+ SGN was co-labeled with tdTomato, suggesting that the cre efficiency was not 100%. Similar results were observed when the whole-mount samples were stained with another neuronal marker Tuj1, and 100% of the tdTomato+ cells were co-labeled with Tuj1 (Fig. 1C). Together, these results demonstrated that the tdTomato reporter specifically labeled the SGNs in Bhlhb5-tdTomato mice.

### Using FACS to isolate the purified Bhlhb5-tdTomato+ SGNs

**Figure 2:**
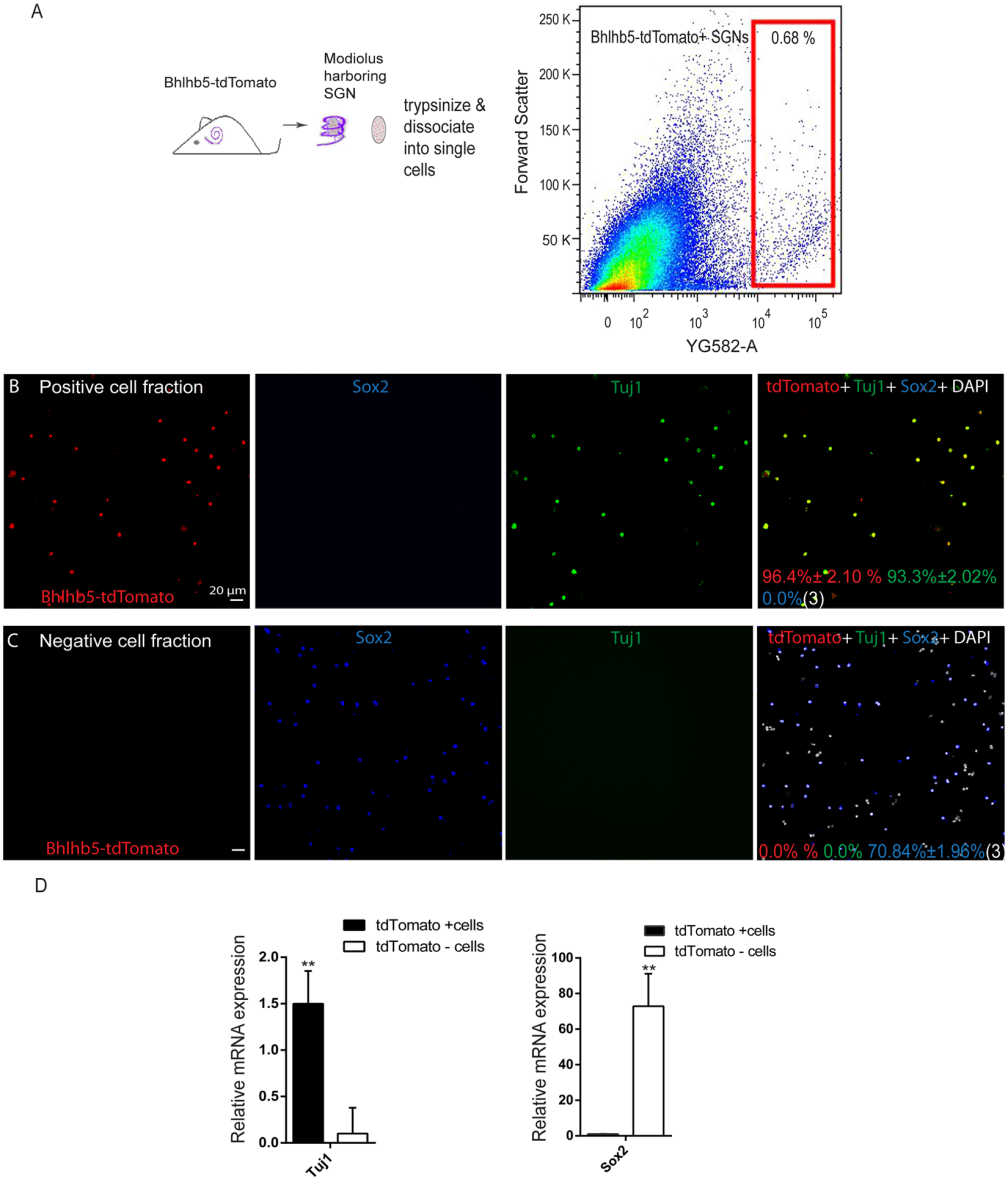
Isolation of Bhllhb5-tdTomato+ SGNs via flow cytometry: (**A**) The modiolus harboring SGNs was dissected from Bhlhb5-tdTomato+ mice and trypsinized to dissociate it into single cells. The tdTomato+ and tdTomato− cells were sorted via flow cytometry. (**B**) Immunostaining of Bhlhb5-tdTomato+ cells after sorting showed a high percentage of Tuj1+ (93.3%) and tdTomato+ (96.4%) cells but no Sox2+ (0.0%) cells. (**C**) Immunostaining of Bhlhb5-tdTomato− cells displayed a high percentage of Sox2+ (97.1%) cells but no Tuj1+ or tdTomato+ cells. (**D**) Quantitative PCR results showed the relatively higher expression of Tuj1 in Bhlhb5-tdTomato+ cells, while the Bhlhb5-tdTomato− cells had relatively higher expression of Sox2. **p < 0.01. Scale bars are 20 μm in (**B** and **C**).

To isolate the Bhlhb5-tdTomato+ SGNs, we genotyped the P3 Bhlhb5-cre+/tdTomato+ mice, dissected out the cochleae, and collected the modiolus harboring the SGNs. After dissociation, we sorted the tdTomato+ cells via flow cytometry from the suspended cells, which made up 0.68% of the viable cells (Fig. 2A). To determine whether the flow-sorted Bhlhb5-tdTomato+ cells were pure SGNs, we performed immunostaining and found that the sorted tdTomato+ cells were 96.4 ± 2.10% tdTomato+, 93.3 ± 2.02% Tuj1+, and 0% Sox2+ (n = 3) (Fig. 2B); while tdTomato− cells were 0% tdTomato+, 0% Tuj1+, and 70.84 ± 1.96% Sox2+ (n = 3) (Fig. 2C). qPCR revealed that the sorted Bhlhb5-tdTomato+ cells had higher expression of Tuj1, while Bhlhb5+/tdTomato− cells showed higher expression of Sox2. These data indicated that the flow-sorted Bhlhb5-tdTomato+ cells were SGNs and that the sorted population of cells was highly pure.

### BMP4 improves the survival of flow-sorted Bhlhb5-tdTomato+ SGNs *in vitro*

**Figure 3:**
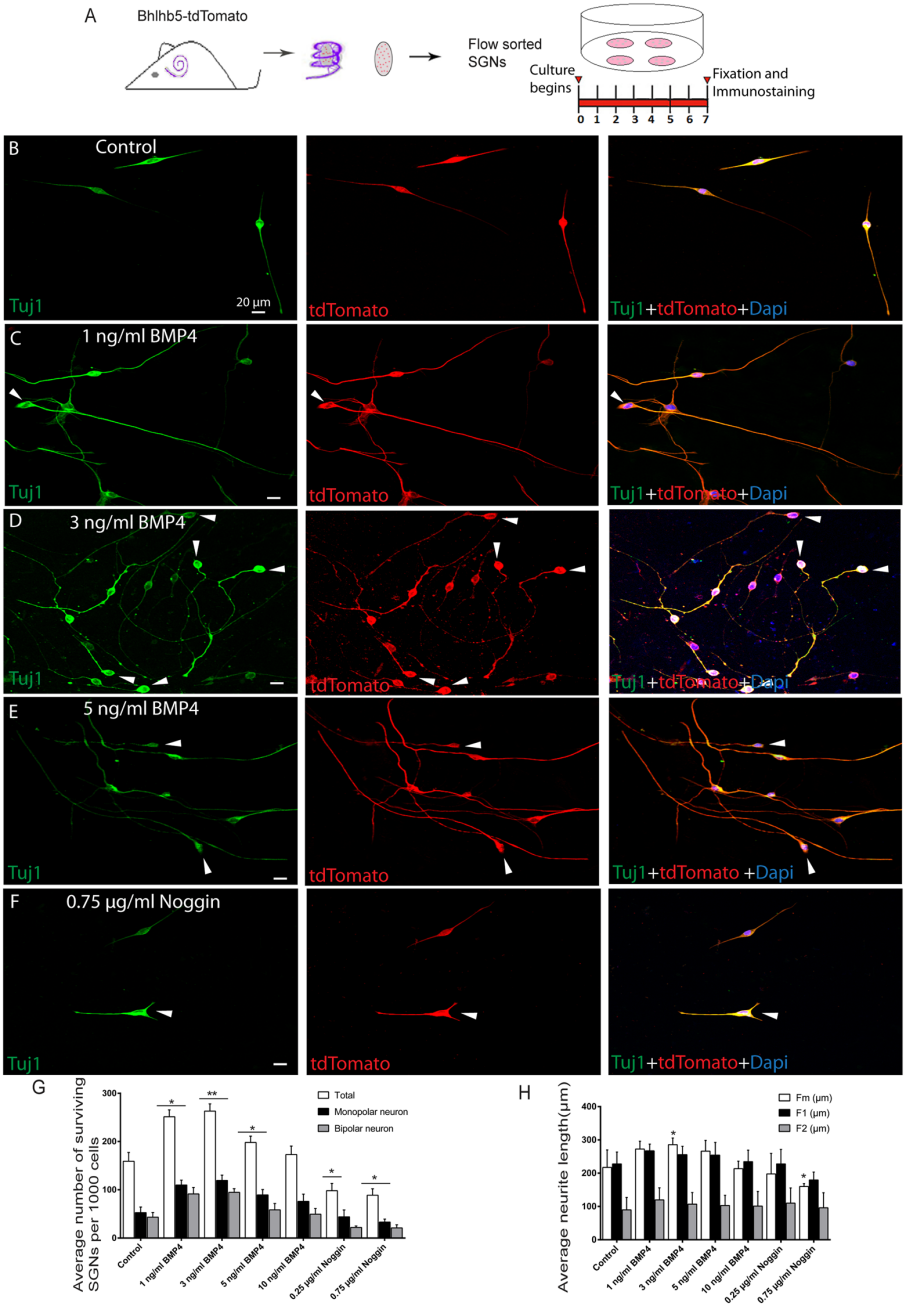
Exogenous BMP4 treatment promoted the survival of flow-sorted Bhlhb5-tdTomato+ SGNs: (**A**) The protocol for how Bhlhb5-tdTomato+ cells from the modiolus were sorted by flow cytometry and cultured for 7 days with different concentration of BMP4 or Noggin. (**B**–**F**) Immunofluorescence imaging of Bhlhb5-tdTomato+ SGNs after treatment with the different concentration of BMP4 or Noggin. There were greater numbers of monopolar neurons (arrowheads) in the BMP4-treated groups compared to the controls, and this number decreased significantly after treatment with Noggin. (**G**) Quantification of the number of surviving SGNs after 7 days of culture. (**H**) Measurement of the neurite lengths extending from the monopolar and bipolar SGNs after 7 days of culture. *p < 0.05, **p < 0.01. Scale bars are 20 μm in (**B**–**F**).

To determine the effects of BMP4 on the survival and outgrowth of flow-sorted Bhlhb5+ SGNs, we cultured 1000 SGNs on laminin-coated 4-well dishes at a density of 10 cells/µl for 7 days in serum-free medium containing different concentrations of BMP4 or Noggin, which is a BMP4 antagonist. Noggin is an extracellular protein that tightly binds to BMP4 and prevents it from binding to the cell surface receptor, thus restricting BMP signaling^44^. We further immunostained the cells with the neuronal marker Tuj1 (Fig. 3A) and found that BMP4 significantly improved the survival of Bhlhb5+ SGNs (Fig. 3B–G). The total number of surviving SGNs in the control condition was 159 ± 10.59 (the numbers of monopolar and bipolar neurons were 52.66 ± 6.69 and 43.33 ± 5.45, respectively). The total numbers of surviving neurons were increased to 251.33 ± 8.21, 263 ± 8.62, 198 ± 7.57, and 173 ± 10.11 with 1 ng/ml, 3 ng/ml, 5 ng/ml, and 10 ng/ml BMP4, respectively (the numbers of monopolar neurons were increased to 110 ± 5.811, 119.34 ± 6.38, 89.33 ± 6.38, and 76 ± 8.71, respectively, and the numbers of bipolar neurons were increased to 91.3 ± 7.79, 94.66 ± 4.37, 58.33 ± 7.68, and 49.33 ± 6.96, respectively) (*p* < 0.05, n = 3) (Fig. 3G). In order to confirm that the increased survival of SGNs was due to the effects of BMP4, we treated cells with the BMP4 antagonist Noggin and found that the total numbers of SGNs were significantly decreased compared to the controls. In the groups treated with 0.25 μg/ml and 0.75 μg/ml Noggin, the total numbers of surviving neurons were decreased to 98.0 ± 8.73 and 88.66 ± 7.79, respectively (the numbers of monopolar and bipolar neurons were decreased to 44 ± 8.18 and 33 ± 3.60 and to 22 ± 1.73 and 21 ± 3.78, respectively) (*p* < 0.05, n = 3) (Fig. 3G). All of these results suggest that BMP4 can promote the survival of cultured cochlear SGNs *in vitro*.

We next investigated the effect of BMP4 on the neurite outgrowth of cultured Bhlhb5+ SGNs *in vitro* by measuring the neurite length of the monopolar and bipolar neurons (Fig. 3B–F and H). We recorded the neurites of bipolar neurons as F1 (the longer neurite) and F2 (the shorter neurite), while the neurite from monopolar neurons was recorded as Fm. We found that the mean Fm length was 217.22 ± 18.43 μm and F1 and F2 were 227.71 ± 18.54 μm and 89.75 ± 9.61 μm, respectively, in the control group. After treatment with different concentration of BMP4, we found that Fm was significantly longer than controls in the 3 ng/ml BMP4 group (285.53 ± 13.10 μm) (*p* < 0.05, n = 3) (Fig. 3H). However, F1 and F2 were only slightly longer than the control group (255.64 ± 19.30 μm and 106.87 ± 9.36 μm in the 3 ng/ml BMP4 group, respectively) (n = 3) (Fig. 3H). When we used Noggin to inhibit the effect of BMP4, we found that Fm was significantly shorter than the control group in the 0.75 μg/ml Noggin group (159.81 ± 12.86 μm) (*p* < 0.05, n = 3) (Fig. 3H). Together, these results suggest that BMP4 promotes the neurite outgrowth of monopolar SGNs.

### BMP4 protects the flow-sorted Bhlhb5+ SGNs against apoptosis

**Figure 4:**
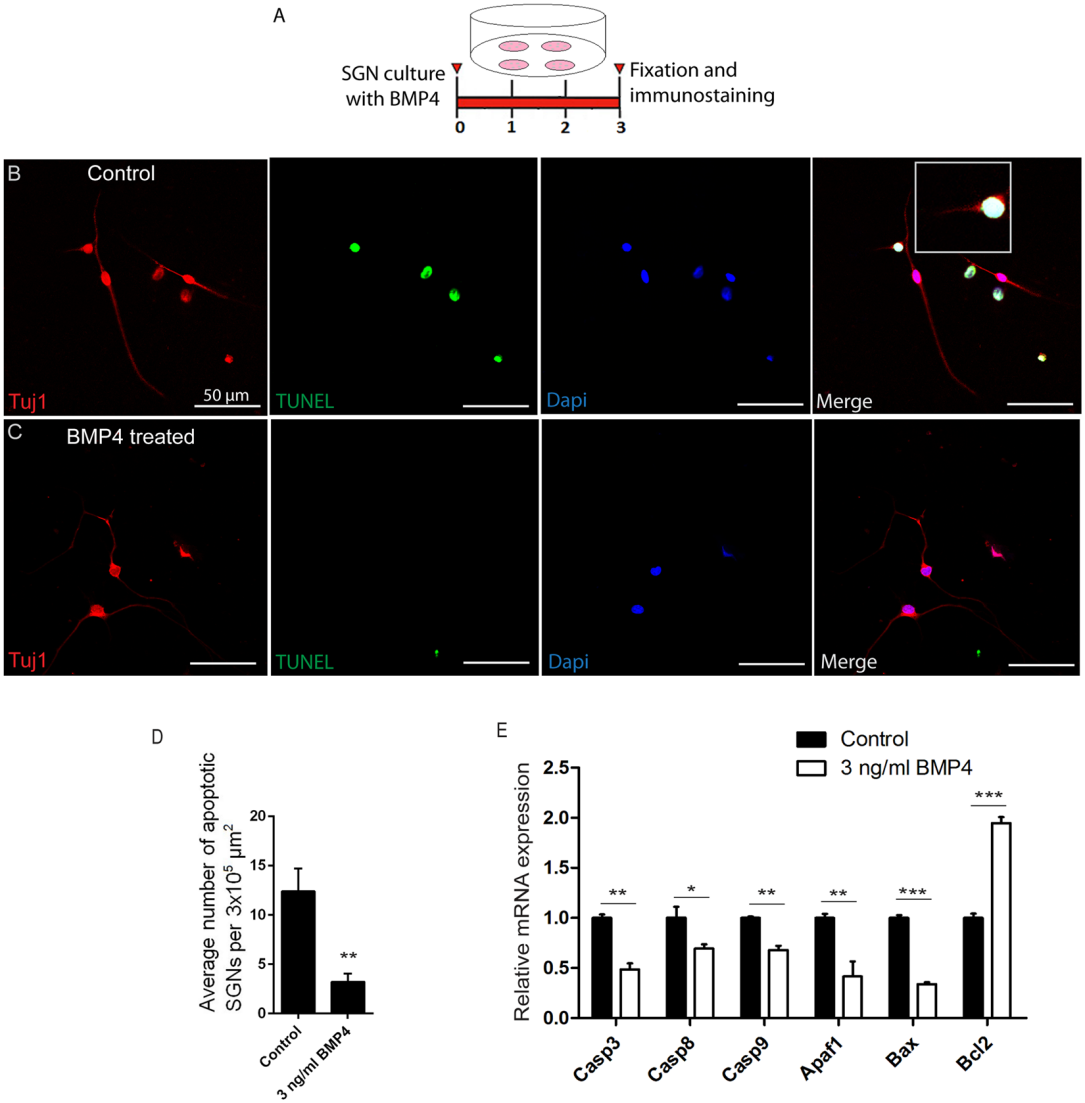
BMP4 treatment protected the Bhlhb5-tdTomato+ SGNs from apoptosis: (**A**) The experimental protocol. (**B** and **C**) There were more apoptotic SGNs observed in the control group as characterized by TUNEL (green), Tuj1 (red), and DAPI (blue) staining. (**D**) In comparison, BMP4 treatment reduced the number of apoptotic SGNs. (**E**) mRNA levels of the apoptosis-related genes were analyzed, and among them the expression levels of five pro-apoptotic genes were significantly reduced while the expression of the anti-apoptotic gene *Bcl2* was significantly increased after BMP4 treatment. **p < 0.01, ***p < 0.001. Scale bars are 50 μm in (**B** and **C**).

To determine the effect of BMP4 on SGN apoptosis, we used the TUNEL assay to detect double-stranded DNA breaks in SGNs^45^. We observed significantly more TUNEL+ apoptotic SGNs in the control group compared to the BMP4-treated groups after being cultured for 72 hours *in vitro* (*p* < 0.01, n = 3) (Fig. 4A–D). Also, the apoptotic SGNs in the control group displayed abnormal morphological characteristics such as cleavage of neurites, compression of neurons, and chromatin condensation. Moreover, qPCR data showed that the BMP4 treatment significantly reduced the expression of pro-apoptotic genes such as *Casp3*, *Casp8*, *Casp9*, *Apaf1*, and *Bax* compared to the control group (*p* < 0.05, n = 3) (Fig. 4E) and significantly increased the expression of the anti-apoptotic gene *Bcl2* (*p* < 0.001, n = 3) (Fig. 4E). These findings indicate that BMP4 protects the SGNs from apoptosis by regulating the expression of apoptosis-related genes.

### Effects of BMP4 on the survival and structure of SGNs in explant culture

**Figure 5:**
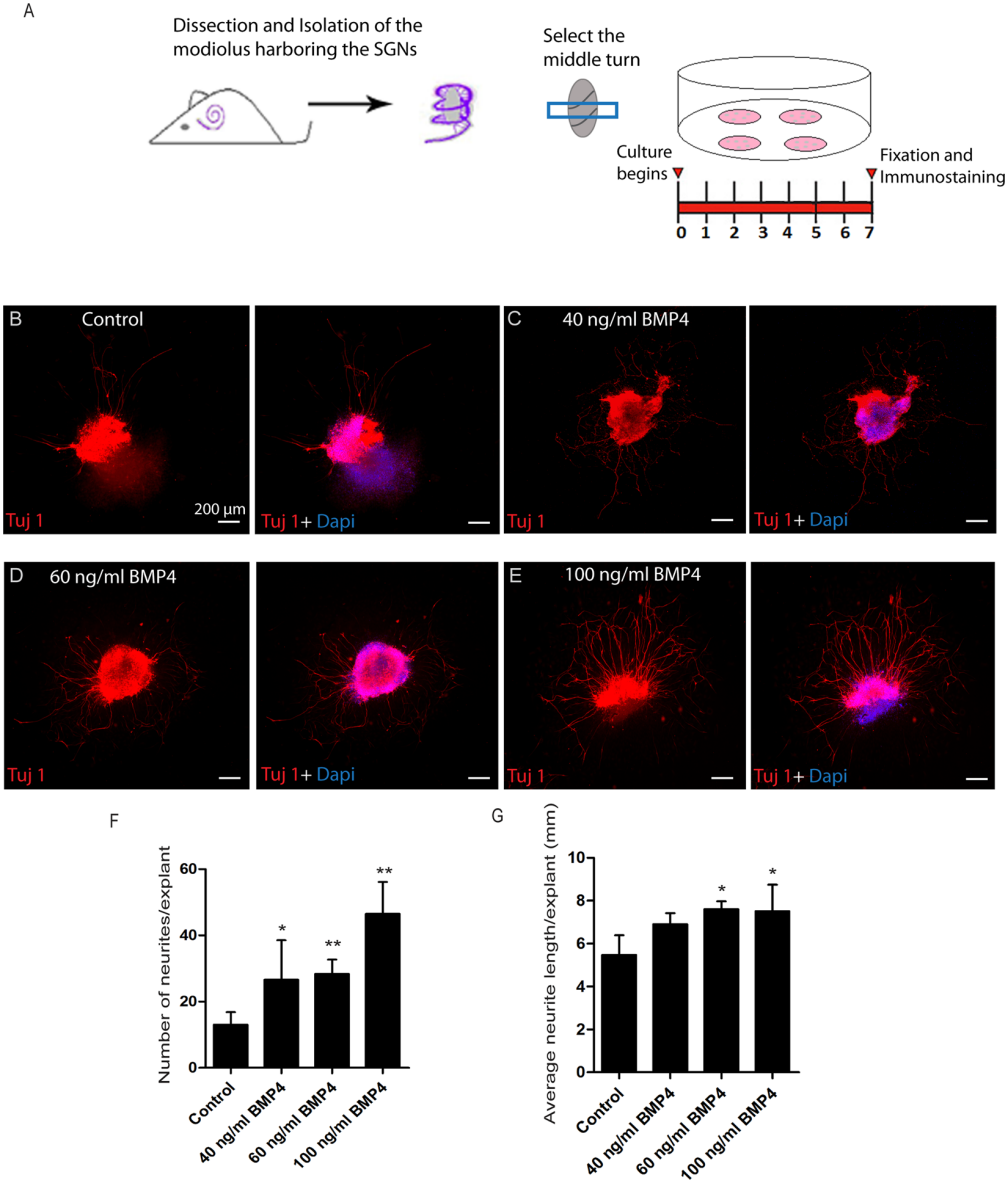
The effects of BMP4 treatment on the survival and outgrowth of SGN explant culture: (**A**) The protocol for the dissection and isolation of the modiolus harboring SGNs. The middle turn was selected for the experiment. (**B**–**E**) Representative immunofluorescence imaging of SGN explant cultures after treatment with different concentrations of BMP4 stained with Tuj1 (red) and DAPI (blue). (**F**) Quantification of the number of neurites per explant. BMP4 treatment significantly promoted the number of neurites per explant. (**G**) Measurement of the neurite length extending from the SGN explant. *p < 0.05, **p < 0.01. Scale bars are 200 μm in (**B**–**E**).

To further investigate the effects of BMP4 on the survival and structure of SGNs in organotypic explant culture, we cultured the middle turn of the modiolus, which contains SGNs, with different concentrations of BMP4 for 7 days (Fig. 5A). We found that BMP4 had a significant effect on the survival of SGNs at high concentrations. When explants were treated with 40, 60, and 100 ng/ml of BMP4, the total numbers of SGNs in each explant were 2.04-, 2.18-, and 3.57-fold greater, respectively, compared to controls (*p* < 0.05, n = 3) (Fig. 5B–F). However, with low concentrations of BMP4 (3, 5, and 10 ng/ml BMP4) we did not observe any significant effects compared to controls (data not shown). High concentrations of BMP4 also preserved the structure and prevented the degeneration of SGNs in cultured explants (Fig. 5G). The average SGN neurite length was also significantly increased at 60 ng/ml and 100 ng/ml of BMP4 (*p* < 0.05, n = 3) (Fig. 5D and E). These data suggest that high concentrations of BMP4 enhance the survival and preserve the structure of SGNs in explant culture *in vitro*.

### Effects of high concentrations of BMP4 on the growth cone of SGNs in explant culture

**Figure 6:**
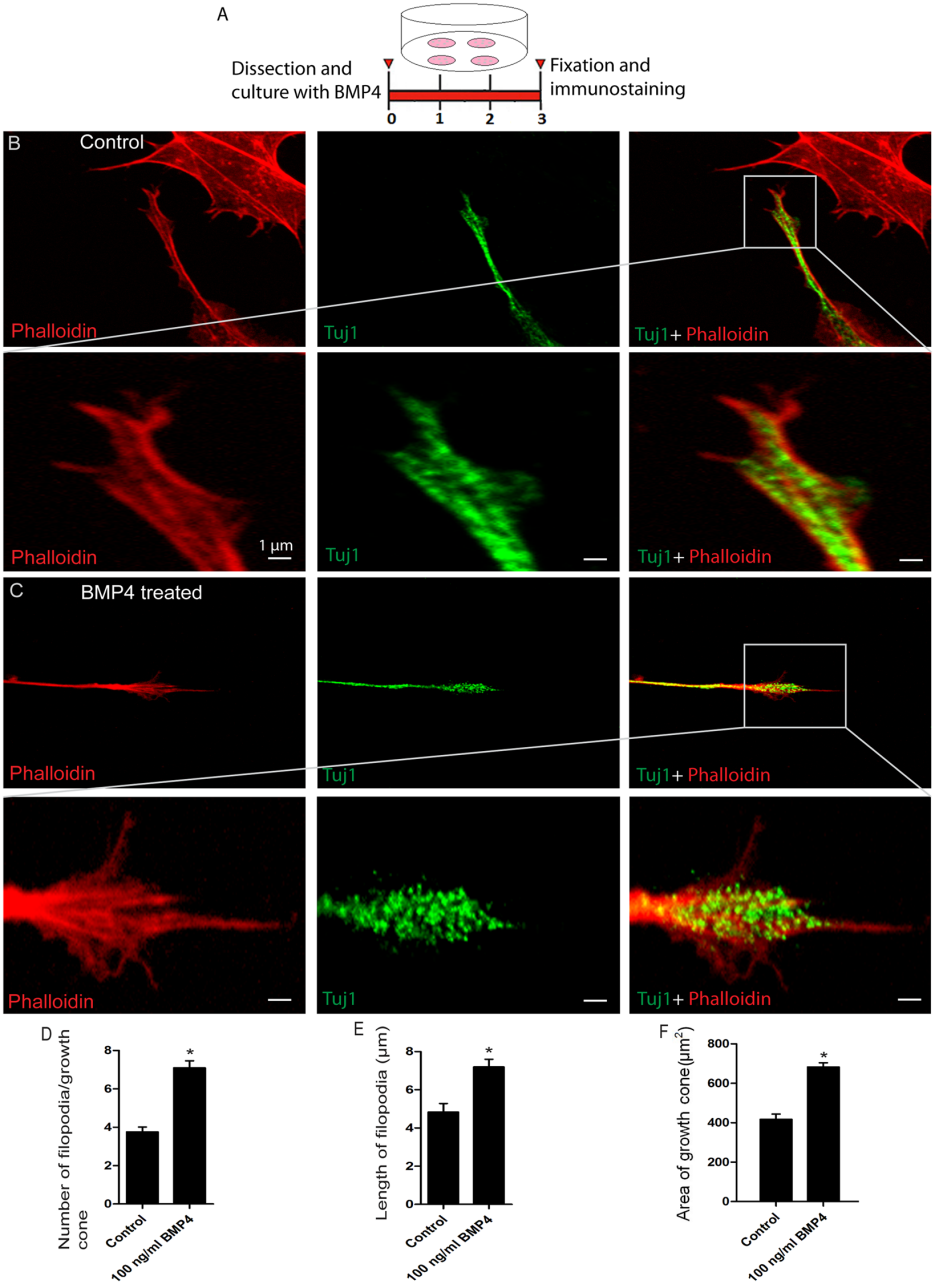
BMP4 treatment influenced the growth of the growth cone in SGN explants: (**A**) The protocol for the experiment. (**B** and **C**) Low and high-magnification immunofluorescence images showing the morphological characteristics of the SGN growth cone after culture with and without BMP4. Phalloidin staining is in red and Tuj1 staining is in green. (**D**) The average number of filopodia per growth cone increased significantly after BMP4 treatment. (**E**) Quantification of the average length of the filopodia. (**F**) The average area of the growth cone. *p < 0.05. Scale bars are 1 μm in (**B** and **C**).

To investigate the effects of BMP4 on the SGN growth cones in explant culture, we dissected the middle turn of the modiolus containing the SGNs, cultured the explants for 72 h, and stained the cultured explants with antibodies against Tuj1 and phalloidin. We took high-resolution images of individual growth cones of SGNs and measured the number and length of filopodia and the area of the growth cone (Fig. 6A). The average number of filopodia originating from the growth cone was significantly greater when treated with 100 ng/ml BMP4 compared to the control group (*p* < 0.05, n = 3) (Fig. 6D). Likewise, the average length of the filopodia and the area of the growth cone were also significantly greater in the 100 ng/ml BMP4 group (*p* < 0.05, n = 3) (Fig. 6E and F). These results indicate that high concentrations of BMP4 promote SGN growth cones compared to the control group.

### High concentrations of BMP4 enhance the synapse density of SGNs in explant culture

**Figure 7:**
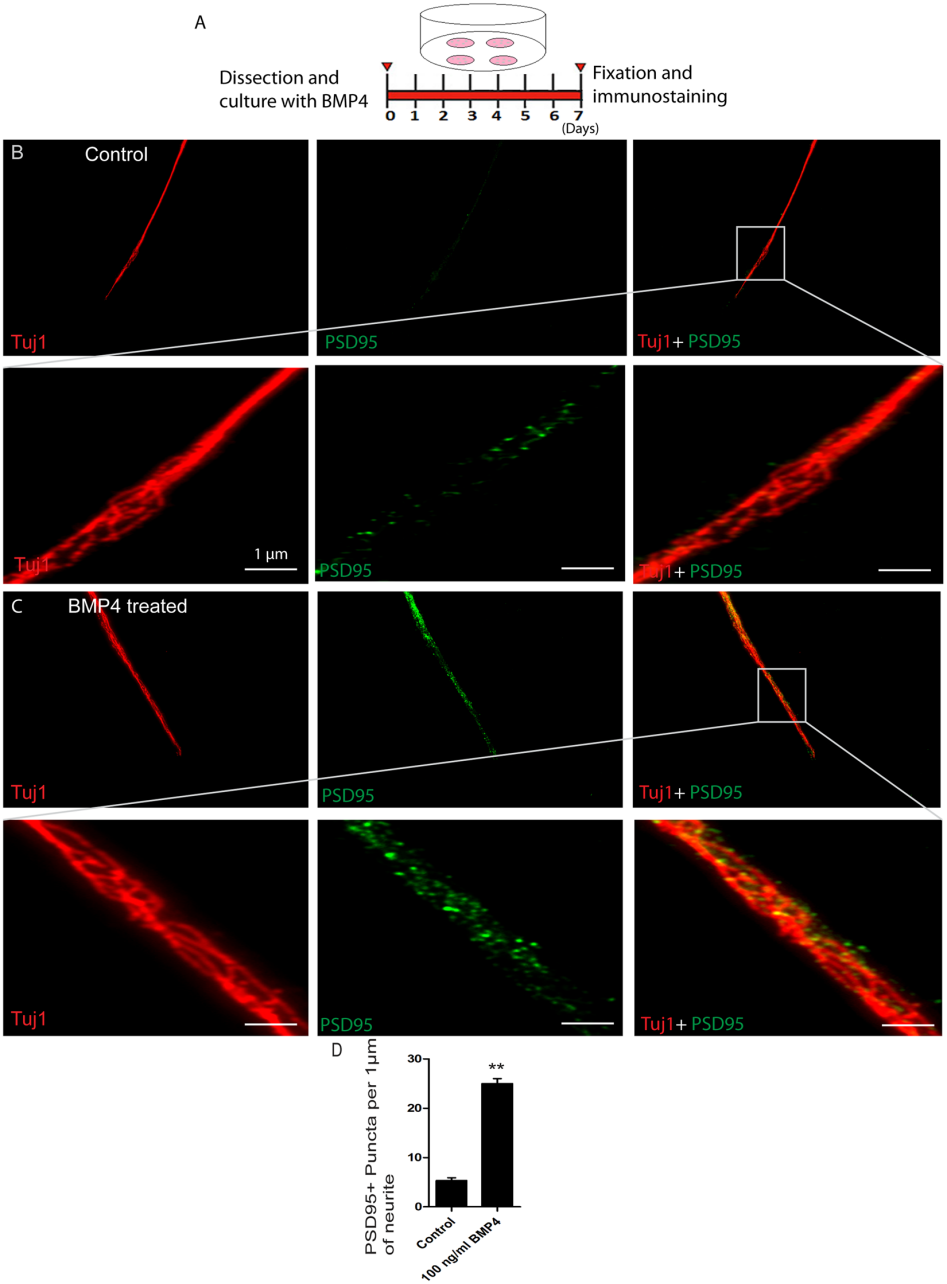
BMP4 treatment enhanced the synapse density in SGN explants: (**A**) The protocol of the experiment. (**B** and **C**) Low and high-magnification representative images showing the synapse density in SGN explants immunostained with PSD95 (green) and Tuj1 (red). (**D**) Counting data showing the number of synapse puncta per 1 µm of neurite in controls and the BMP4-treated group. *p < 0.05. Scale bars are 1 μm in (**B** and **C**).

To further examine the effects of BMP4 on the function of SGNs in explant culture, we measured the expression of the synapse marker PSD95 in SGN neurites as an indication of synapse density after 7 days of culture (Fig. 7A). PSD95 is a postsynaptic protein that participates in synapse maturation and synaptic plasticity^46, 47^. We found that the explants treated with 100 ng/ml BMP4 had significantly elevated synapse density compared to the control group (Fig. 7B and C). We measured the number of PSD95+ puncta per 1 µm of neurite length and found that the puncta density was significantly increased in the 100 ng/ml BMP4 cultures (*p* < 0.01, n = 3) (Fig. 7D), suggesting that high concentrations of BMP4 promote the synapse formation and synaptic plasticity of SGNs in organotypic explant culture.

## Discussion

The most common approach to studying SGNs has been the dissection, isolation, and dissociation of the modiolus prior to *in vitro* culture^37, 41, 48^. However, this method of whole modiolus dissociation means that one cultivates all cell types in the modiolus, including SGNs, glial cells, and fibroblasts, thus this method suffers from the inability to specifically study a pure population of SGNs. In particular, it is impossible to isolate SGNs from the modiolus using a stereoscopic dissecting microscope because of the interconnected network of SGNs with supporting cells such as glial cells and fibroblasts. Thus, SGN isolation and culture using this method primarily allows one to study the cell-cell interactions of different population of cells in the modiolus but does not allow one to specifically study SGNs in culture.

In this study, we have established a method to successfully isolate a pure population of SGNs via FACS. We used a Bhlhb5-cre+/tdTomato+ transgenic mouse model to label SGNs, and immunofluorescence labeling of cryosections and whole-mount modiolus explants showed that tdTomato+ cells located in the modiolus were co-labeled with specific neuronal markers such as anti-NeuN and anti-Tuj1 antibodies. Due to the limits of cre efficiency, some of the NeuN+ and Tuj1+ SGNs did not express the tdTomato reporter; however, every tdTomato+ cell was also NeuN+ and Tuj1+ suggesting that the tdTomato+ cells are a pure population of SGNs. In addition, the population of tdTomato+ cells isolated through FACS had higher levels of *Tuj1* mRNA expression and lower levels of *Sox2* mRNA expression demonstrating that the flow-sorted population of SGNs was highly pure. Conversely, the greater mRNA expression level of *Sox2* in tdTomato− cells showed that these cells contain large population of glial cells.

A recent study by Schwieger *et al*. demonstrated a method to establish an *in vitro* SGN culture without supporting cells by adding cytarabine to the *in vitro* culture system, which decreased the proliferation of non-neuronal cells such as glial cells by inhibiting their mitotic division^48^. However, cytarabine is a chemotherapeutic drug and has been reported to have neurotoxic effects^49–51^. Thus, our use of a transgenic mouse model to specifically label and isolate a pure population of SGNs via FACS might be a better method for performing *in vitro* experiments on SGNs.

BMP4 has been shown to participate in neuronal survival in the central nervous system. Peripherally derived BMP4 promotes the survival of embryonic motor neurons and protects against glutamate-induced excitotoxicity *in vitro* ^52^. In the gut wall, BMP4 regulates the migration of enteric nervous system precursors, promotes ganglion cell aggregation, and induces significant neurite fasciculation^53^. Several studies have shown the expression of different BMPs such as BMP2, BMP3, BMP4, BMP5, and BMP7 during the embryonic and postnatal development of the mouse and chicken inner ear^25, 54–56^. BMP4 is expressed in the developing otic placode at E9.0, and it continues to be expressed in the sensory epithelium and mesenchyme until P1^57^. It regulates the epithelial-mesenchymal tissue interaction that is responsible for the differentiation and establishment of the cartilaginous otic capsule in the inner ear^58^. BMP4−/− knockout mice die between E6.5 and E9.5, while BMP4+/− heterozygous mice are viable to adulthood but exhibit circling behavior due to defects in the inner ear^38, 59^. The auditory brainstem response of BMP4+/− mice shows elevated hearing thresholds and reduced numbers of SGNs in the organ of Corti suggesting that BMP4 is crucial for the development of the auditory organ^33^. Our previously published work has shown that when embronic cochlear sensory epithelium are cultured with Noggin *in vitro*, Sox2+ cells are markedly reduced and inner ear progenitors fail to differentiate into hair cells, suggesting that BMP signaling is required for cochlear sensory cell fate decision^29^. Takahiro *et al*. proposed a model in which a restricted domain of Bmp4 expression imparts patterning information to sensory precursor cells in the cochlear primordium from E11.5 onward and that an asymmetric BMP signaling gradient is key to inducing and patterning the sensory domains of the mammalian cochlea^28^. In *in vitro* chick otocyst cultures, exogenous BMP4 significantly increases the number of sensory hair cells, while blocking BMP signaling reduces the number of supporting cells and hair cells^27^. Similarly, when culturing chick statoacoustic ganglion explants, BMP4 provides guidance cues for the growing axon and promotes the growth of statoacoustic ganglion neurons^60^. In another *in vitro* avian otocyst study, Gerlach *et al*. used agarose beads combined with cells expressing BMP4 or Noggin^61^. Their results showed that although the BMP4-producing cells had no effect on the mature inner ear structure when implanted alone, Noggin-producing cells implanted adjacent to the BMP4 cell foci prevented normal semicircular canal development, which means a different role for BMP4 in the accurate and complete morphological development of the semicircular canals. Our results show that exogenous treatment with BMP4 promotes the survival of flow-sorted Bhlhb5+ SGNs and enhances the neurite outgrowth of monopolar neurons when compared with the control group. In comparison with the previous work of Whitlon *et al*. who reported the increased survival of SGNs after applying a combination of BMP4, BDNF, and NT3 in their *in vitro* culture system^37^, we used culture medium without any other growth factors as the control group. In a dose-response experiment, we found that the 3 ng/ml concentration of BMP4 significantly promoted the survival of cultured Bhlhb5+ cochlear SGNs and enhanced the neurite outgrowth of monopolar neurons; however, the higher concentrations of BMP4 did not further promote the survival of Bhlhb5+ cochlear SGNs, which might be due to the saturation or downregulation of BMP receptors because this kind of dose response has also been observed for other growth factors^62^. We also found that BMP4 reduced the number of apoptotic SGNs and led to significantly lower expression of pro-apoptotic genes and higher expression of anti-apoptotic genes compared to controls.

BMP4 plays many critical roles during the development of the central nervous system, and it continues to regulate the differentiation of neural stem cells into neurons, astrocytes, and oligodendrocytes in the adult central nervous system^63^. It stimulates the neuronal differentiation of neural stem cells through the activation of the extracellular signal-related kinase (ERK) pathway, and it induces the expression of the neuron-specific class II beta tubulin^64^. BMP4 has also been found to induce the differentiation of human embryonic stem cells into sensory neurons that express neuronal markers such as beta tubulin III and peripherin^65^, indicating that in the inner ear BMP4 might also play roles in axonal guidance and the formation of SGN synapses. A previous study reported that BMP4+/− heterozygous mice exhibit reduced cochlear innervations, suggesting that BMP4 is an essential factor for normal cochlear hair cell innervation, and the absence of such innervation is associated with partial deafness^33^.

The organotypic culture of SGNs has been commonly used to investigate the effects of different growth factors on SGNs^66–69^. We investigated the effects of BMP4 on SGN explants, and compared to the single-cell culture we observed that low concentrations of BMP4 did not have any effects on SGN explants, which might be due to the complex biological structure of the SGN explant that might prevent BMP4 from reaching the SGNs. However, we found that high concentrations of BMP4 led to the significant preservation of the number of neurites extending from the explant and increased the length of the neurites, suggesting that BMP4 promotes the survival and structure of SGNs in explant culture. Moreover, after being cultured with high concentrations BMP4, the number and length of filopodia and the area of the growth cones were significantly increased compared to the control group. We also observed enhanced expression of the postsynaptic marker PSD95 in the high-concentration BMP4 treatment, demonstrating that BMP4 significantly elevated the synapse density of SGNs. However, it remains unclear how BMP4 exerts its different effects at the different developmental stages of the mouse cochlea in vivo, and there is no evidence or research about the normal role of BMP4 in the mature cochlea, which should be the focus of future research.

In summary, we have demonstrated a new method to isolate pure populations of SGNs by using the Bhlhb5-cre+/tdTomato+ transgenic mouse model, and we have demonstrated that BMP4 promotes the survival of flow-sorted Bhlhb5+ SGNs and protects them against apoptosis. In particular, BMP4 promotes the outgrowth of monopolar neurites. Furthermore, we demonstrated that high concentrations of BMP4 preserve the survival and structure of SGNs and facilitate the neurite outgrowth and synapse plasticity of SGNs in explant culture. Taken together, our findings provide a more sophisticated way to isolate pure populations of SGNs and demonstrate that BMP4 might hold the potential to protect cochlear SGNs and preserve the structure and function of SGNs *in vitro*.
